# O9.6. SPECIFIC SYMPTOMS IN ADOLESCENCE PREDICT PSYCHOSIS IN THE NORTHERN FINLAND BIRTH COHORT 1986

**DOI:** 10.1093/schbul/sby015.250

**Published:** 2018-04-01

**Authors:** Juha Veijola, Tuula Hurtig, Graham Murray, Pirjo Mäki

**Affiliations:** 1 University of Oulu; 2 University of Cambridge

## Abstract

**Background:**

A number of psychological symptoms have been found to predict psychosis. Many studies have found no specificity to separate symptoms predicting non-psychotic psychiatric disorders from those predicting psychotic disorders. We were able to conduct prospective study comparing adolescent symptoms predicting non-psychotic psychiatric disorders and psychotic psychiatric disorders.

**Methods:**

Members of the of the Northern Finland Birth Cohort 1986 were asked to fill in PROD-screen questionnaire at age 15–16 years. PROD-screen includes 21 items both measuring positive prodromal symptoms, negative prodromal symptoms and general symptoms.

We were able to follow 6,514 participants using Finnish Hospital Discharge Register detecting new hospital treated mental disorders till 23 years.

**Results:**

The highest prevalence of positive symptoms in the PROD-screen were in the group of subjects who developed psychotic disorder (65% over the cut off) compared to subjects who developed non-psychotic disorder (36%; OR 5.7; 95%CI 2.1–15.4, p<0.001, adjusted for parents’ psychiatric disorder, family structure, family SES, adolescent’s cannabis use and gender), and to subjects without any disorder (27%; adjusted OR 6.5; 2.8–15.0, p<0.001). Respective figures for negative symptoms were 55% in the group of psychotic subjects compared to 30% in subjects with non-psychotic disorder (3.3; 1.4–7.7, p=0.01) and 24% in the ‘healthy’ (4.1; 1.9–8.6, p<0.001).

When comparing separate symptoms in those having psychiatric hospital treatments, we found four positive symptoms and one negative symptom predicting specifically psychotic disorders.

**Discussion:**

In this large prospective population sample both positive and negative symptoms in adolescence associated specifically with development of first episode psychosis.

